# Supplementary material for: Overcoming Asymmetric Contact Resistances in Al-Contacted Mg2(Si,Sn) Thermoelectric Legs
Source: Materials (Basel). 2021 Nov 10;14(22):6774. doi: 10.3390/ma14226774 (PMC8623178; doi:10.3390/ma14226774)
Supplement: Supplementary file 1 [file materials-14-06774-s001.zip › materials-1431542-supplementary.pdf]

materials

Supplementary Information

Overcoming Asymmetric Contact Resistances in Al-Contacted $Mg_2(Si,Sn)$ Thermoelectric Legs

Julia Camut [1,*], Sahar Ayachi [1], Gustavo Castillo-Hernandez [1], Sungjin Park [2], Byungki Ryu [2], SuDong Park [2], Adina Frank [1], Christian Stiewe [1], Eckhard Mueller [1,3] and Johannes de Boor [1,4,*]

[1] Department of Thermoelectric Materials and Systems, Institute of Materials Research, German Aerospace Center, 51147 Cologne, Germany;
[2] Korea Electrotechnology Research Institute (KERI), Energy Conversion Research Center, 12, Jeongiui-gil, Seongsan-gu, Changwon-si 51543, Gyengsangnam-do, Korea;
[3] Institute of Inorganic and Analytical Chemistry, JLU Giessen, 35390 Giessen, Germany
[4] Institute of Technology for Nanostructures (NST), Faculty of Engineering, University of Duisburg-Essen 47057 Duisburg, Germany
* Correspondence: julia.camut@dlr.de (J.C.); johannes.deboor@dlr.de (J.d.B.)

Citation: Camut, J.; Ayachi, S.; Castillo-Hernandez, G.; Park, S.; Ryu, B.; Park, S.; Frank, A.; Stiewe, C.; Mueller, E.; de Boor, J. Overcoming Asymmetric Contact Resistances in Al-Contacted $Mg_2(Si,Sn)$ Thermoelectric Legs. *Materials* **2021**, *14*, 6774. https://doi.org/10.3390/ma14226774

Academic Editor: Takao Mori

Received: 6 October 2021
Accepted: 5 November 2021
Published: 10 November 2021

Publisher's Note: MDPI stays neutral with regard to jurisdictional claims in published maps and institutional affiliations.

Copyright: © 2021 by the authors. Licensee MDPI, Basel, Switzerland. This article is an open access article distributed under the terms and conditions of the Creative Commons Attribution (CC BY) license (https://creativecommons.org/licenses/by/4.0/).

Studying the asymmetry of the electrical contact resistivities

The phenomenon of asymmetry in electrical contact resistances has been observed with other electrodes than aluminum, as can be seen in Table S1. Although the magnitudes are different, this behavior also appears with Ni and Ag electrodes on Mg_2X materials. The Ag electrodes were contacted at 450 °C and 30 MPa for 10 min and the Ni electrodes at 600 °C and 30 MPa for 10 min. This shows that this issue is not specific to Al and can even be encountered with electrodes that are less prone to oxidation.

Table S1. Asymmetric electrical contact resistivities for several electrodes on Mg_2X materials.

Electrode/TE	Symmetric sample r_c (μΩ·cm²)	Asymmetric sample (low // high r_c) (μΩ·cm²)
Al/Mg_2(Si,Sn)	4 ± 2	15 ± 19 // 792 ± 181
Ni/Mg_2Si	4 ± 3	7 ± 7 // 179 ± 116
Ag/Mg_2(Si,Sn)	9 ± 1	9 ± 5 // 40 ± 36

Table S2 also shows cases of symmetric and asymmetric samples for various factors, such as the carrier type, TE pellet geometry (diameter of 15 or 30 mm), presence or absence of a buffer layer during contacting and direct vs indirect current heating setup. The presence of both symmetric and asymmetric samples for all configurations of these factors indicates that they do not seem to play a decisive role in this asymmetry phenomenon.

Table S2. List of symmetric and asymmetric samples with varying contacting parameters.

Sample	Low r_c (μΩ·cm²)	High r_c (μΩ·cm²)	Carrier type	Pellet diameter (mm)	Direct/ Indirect current	Buffer layer
1119JCA04	3 ± 2	symmetric	n	15	indirect	yes
1119JCA78	18 ± 7	220 ± 180	n	15	indirect	yes
1119JCA20	54 ± 26	107 ± 40	n	30	indirect	yes
1118NT20	5 ± 4	Symmetric	n	30	indirect	yes
1118NT71	7 ± 2	98 ± 13	p	15	indirect	yes
1118NT76	11 ± 4	symmetric	p	15	indirect	yes
1117NP33	9 ± 5	symmetric	p	15	indirect	no
1119JCA86	4 ± 2	Symmetric	n	15	direct	no
1120JCA09	49 ± 27	319 ± 318	n	15	direct	no

EDX line scans at the Al-TE interface of a symmetric and an asymmetric sample are shown in Supplementary Figure S1 and the respective contact resistivities are reported in Table S3. Both samples went through the same preparation and process steps. Although the resolution of the images is not optimal due to a deficient polishing preparation, the EDX line scans show oxygen peaks at the interfaces of the asymmetric sample, with a rough correlation between peak height and r_c. Such peaks are not observed on the symmetric sample. The O peak could correspond to a very thin Al_2O_3 layer, which cannot be distinguished on the curves due to the scale.

Al_2O_3 could indicate that there was an oxide layer on the foils of the asymmetric sample before contacting and could be the origin of the asymmetry. Given that both samples were identically prepared and processed, this would indicate how variable and non-reproducible the manual foil preparation is. It is also possible that the Al_2O_3 was formed after contacting and dicing, as the dicing could have pulled on the interface or cracks could have nucleated, making a way for oxygen to diffuse at the interface and oxidize the Al electrode.

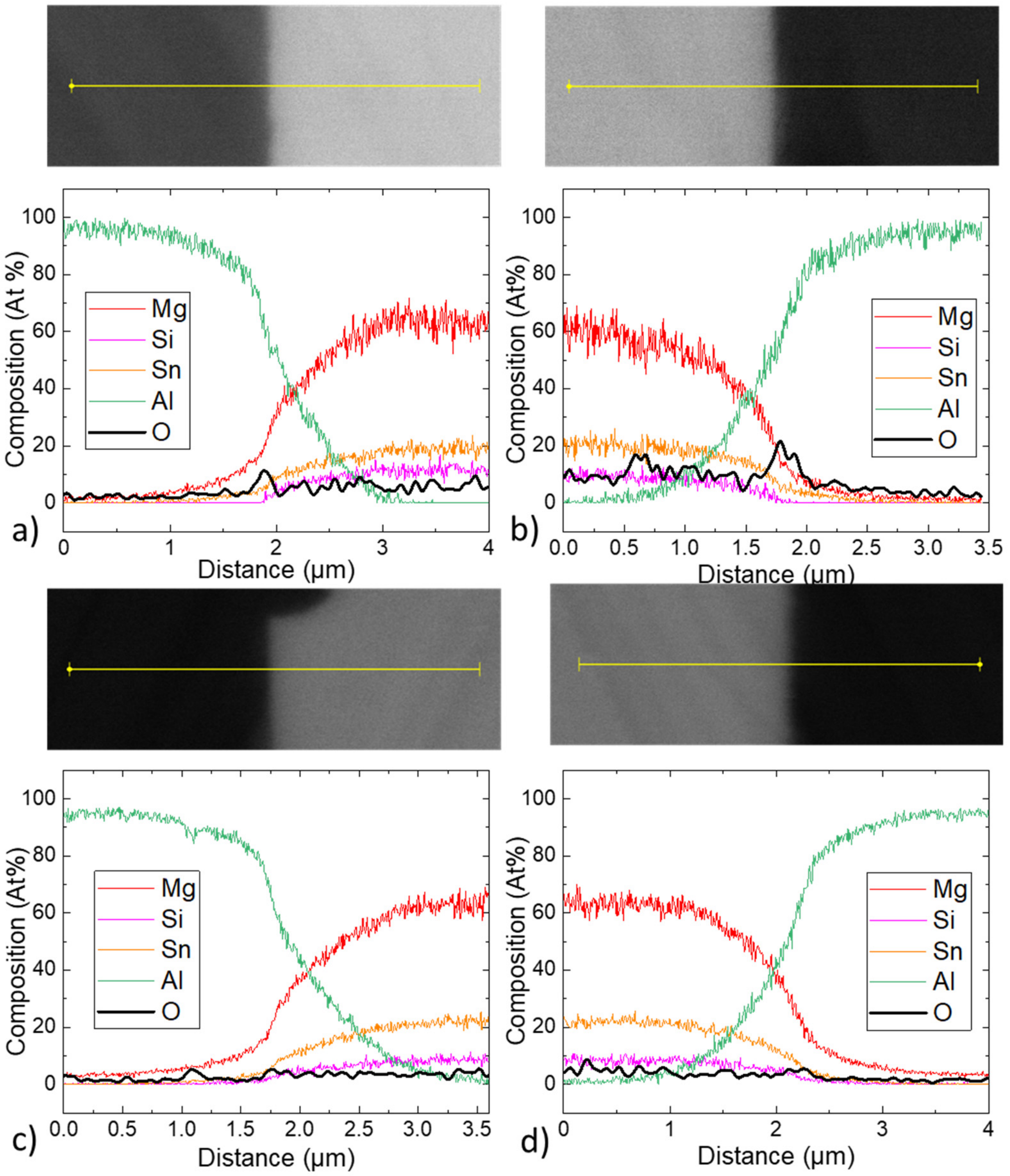

Figure S1. Linescans of Al/Mg_2(Si,Sn) interfaces. (**a**) left of sample 1, (**b**) right of sample 1, (**c**) left of sample 2, (**d**) right of sample 2.

Table S3. - Electrical contact resistivities of the Al/Mg_2(Si,Sn) interfaces presented in Figure S1.

**interface**	**r_c value (μΩ·cm²)**
a	5 ± 19
b	792 ± 181
c	4 ± 3
d	4 ± 3

Zn coating and contacting experiment

Figure S2 shows SEM images of the Zn coating on the Al foil after ion etching in the PVD. The Zn layer looks dense and continuous and is ~8 μm thick. It should therefore ensure an efficient protection against air to the Al foil. Figure S3 shows a picture of the contacted pellet. It is seen that a large proportion of the Zn melted and got evacuated on the sides of the pellet due to the applied pressure.

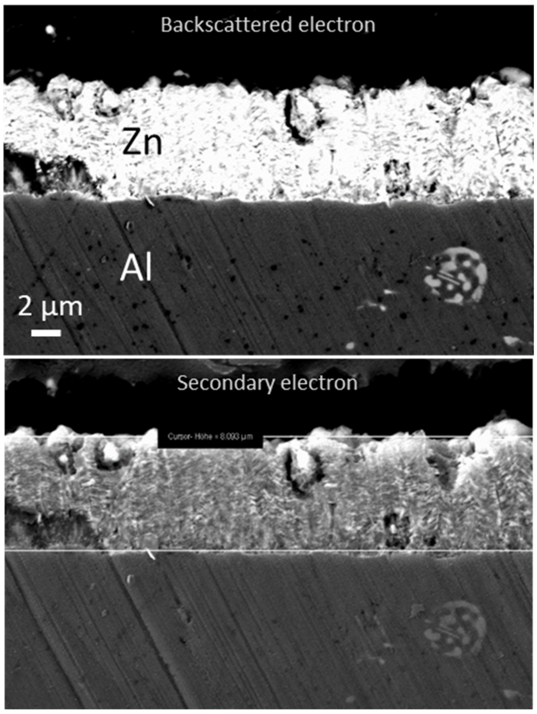

Figure S2. BsE and SE2 observations of the Zn coating on the Al foil after ion etching.

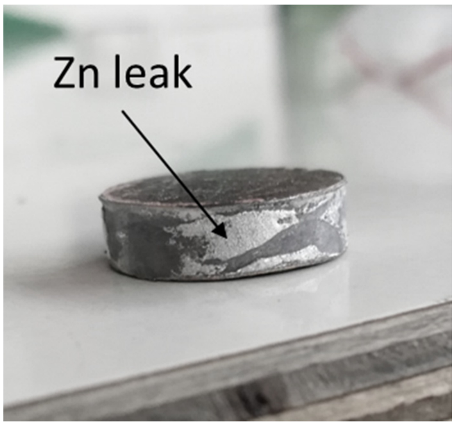

Figure S3. picture of a $Mg_2(Si,Sn)$ pellet after contacting with Zn-coated electrodes.

Figure S4 shows exemplary line scans of the samples presented in Tables 1, 2 and 3 of the paper. As each pellet was cut into 9 legs, and a full line scan characterization is very lengthy, most legs were measured with local line scans which focus on each interface on a scale of 1–2 mm, separately. This is the case of the top/bottom leg presented below (Figure S4a and b).

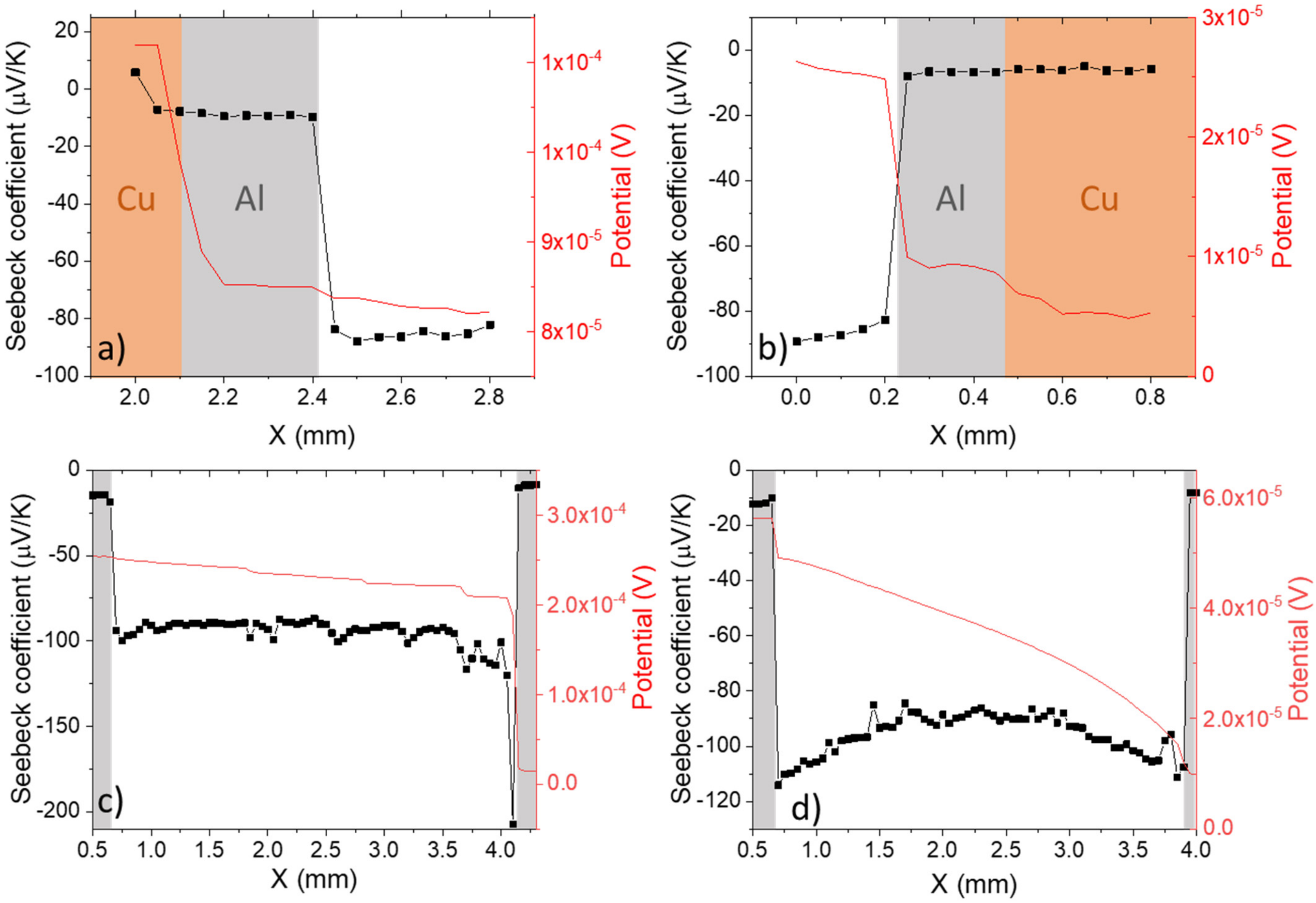

Figure S4. Exemplary line scans of the potential and Seebeck coefficients for the reported experiments (**a**) top/bottom experiment, side at the top during cutting (leg7), b) top/bottom experiment, side at the bottom during cutting (leg 7), c) top/top experiment (leg 4), d) experiment with Zn-coated Al foils (leg 5). The Cu layer indicated in a) and (**b**) corresponds to the sample holder of the PSM.

Additional hybrid-DFT calculations

In order to compare with experimental results of the Zn contacting experiments, hybrid-DFT calculations were made to investigate the defects formation energies of Zn-related defects in Mg_2X systems. Figure S5 shows the defect formation energy calculations in Li and Bi-doped Mg_2Si. Bi-doped samples are discussed under both Mg-poor and Mg-rich conditions, while for Li-doping only the Mg-poor conditions are discussed. Indeed, Li doping is aimed at Mg sites in the Mg_2X material while it was found that, although Li on Mg site defects are indeed stable, Li interstitials are even more stable defects [1]. This means that the Li-doped material will always contain a certain proportion of Mg vacancies and Mg-rich conditions will not be obtained. In Figure S5, only the defects with formation energies < 1 eV are presented.

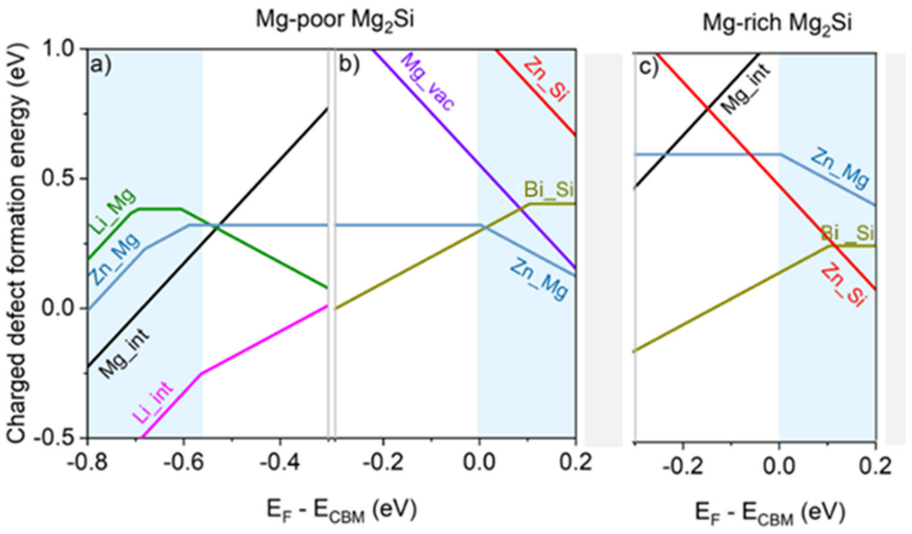

Figure S5. Charged defect formation energies for Zn defects in: (**a**) Li-doped and (**b**) Bi-doped Mg_2Si under Mg-poor conditions (**c**) Bi-doped Mg2Si under Mg-rich conditions, (**d**) Li-doped Mg_2Sn under Mg-poor conditions. For simplicity, the Li-doped Mg_2Si and Mg_2Sn are only represented in the Fermi level region around VBM while the Bi-doped samples are represented in the Fermi level region around CBM.

In Supplementary Figure S5a where the results of Li-doped Mg_2Si are presented, the most relevant Zn defect in the vicinity of the valence band maxima (VBM) is Zn_{Mg} (Zn_Mg). This defect has a charge transition level (CTL) from q = 1+ to q = 0 at E_F = − 0.59 eV, below the VBM. For E_F between −0.68 and −0.59 eV where Zn_{Mg} is a donor defect with the charge q = 1+, the electron contribution to the charge carrier concentration is not expected to be very significant, owing to its high formation energy (E_{form} = 0.22 ~ 0.32 eV) compared to other more stable donor defects, namely I_{Li} (Li_int) and I_{Mg} (Mg_int). Nevertheless, as the intendent conduction here is p-type, the occurrence of the Zn_{Mg} defect would make it even harder to achieve such conduction, as it is yet another donor defect with a formation energy lower than that of Li_{Mg} (Li_Mg), which is the major acceptor defect.

For Bi-doped Mg_2Si under Mg-poor conditions, as presented in Supplementary Figure S5b, the relevant Zn defects are Zn_{Mg} and Zn_{Si} (Zn_Si). Zn_{Mg} is a neutral defect across the band gap and has a charge transition level above the conduction band minima (CBM) (at E_F = 0.003 eV). Above this CTL, Zn_{Mg} becomes an acceptor defect of charge q = 1-. If we take the example of highly doped sample with E_F = 0.05 eV, one can see that the main electron donor defect Bi_{Si} and the Zn_{Mg} defect have very comparable formation energies: 0.35 eV for the former, 0.27 eV for the latter. Using $n\,(D^q)=\theta_{\mathrm{deg}} n_{\mathrm{latt}} \exp(-\frac{E_{\mathrm{form}}}{k_B T})$ where k_B = 8.62 × 10^{-5} eV/K, n_{latt}= 1.6 × 10^{22} cm^{-3} for a defect on Si or Sn site in Mg_2Si and 3.1 × 10^{22} cm^{-3} for a defect on Mg sites and T = 475 °C, the defect density for the Bi_{Si} (Bi_Si) and the Zn_{Mg} defects would respectively be 7.0 × 10^{19} cm^{-3} and 4.7 × 10^{20} cm^{-3}, which corresponds to a compensation in n ~ 4 × 10^{20} cm^{-3}. Therefore, Zn_{Mg} is expected to cause a significant compensation of the conduction electrons provided by Bi_{Si} for the Mg_2Si system under Mg poor conditions. Experimentally, this would translate to a variation in the Seebeck coefficient.

As for the second Zn-related defect in this case, Zn_{Si}, it is not expected to play an important role in determining the system´s carrier concentration because its high formation energy results in a low density. Therefore, the effect of the Zn_{Si} defect can be disregarded.

For Bi-doped Mg_2Si under Mg-rich conditions, as presented in Supplementary Figure S5c, the relevant Zn defects are also Zn_{Mg} and Zn_{Si}, however with different formation energies. Zn_{Mg} is a much less stable defect than under Mg-poor conditions with E_{form} = 0.61

eV at CBM. Therefore, it is not expected to notably influence the charge carrier concentration. On the other hand, the Zn_{Si} defect is much more stable under Mg-rich (Si-poor). Zn_{Si} is an acceptor defect of charge q = 2- in the whole chemical potential of interest region. It has a formation energy E_{form} = 0.39 eV at E_F = 0.05 eV. At this second E_F region, the formation energies of Zn_{Si} and Bi_{Si} are close, and get closer the higher the energy level. Bi_{Si} has a formation energy E_{form} = 0.20 eV at E_F = 0.05 eV. If we calculate the defect density for both defects, it would be 3.8 × 10^{19} cm^{-3} for Zn_{Si} and 7.2 × 10^{20} cm^{-3} for Bi_{Si}, which corresponds to a compensation in n ~ 7 × 10^{20} cm^{-3}. Experimentally, a variation in the carrier concentration would be expected under Mg-rich conditions.

As a conclusion, the predicted changes in carrier concentration due to Zn diffusion are similar for both Mg_2Si and Mg_2Sn. For both systems, the most stable Zn-related defect is Zn_{Mg} for Mg-poor conditions and Zn_X (X=Si or Sn) for Mg-rich conditions and all are electron killers in the n-type materials. As a consequence, the observed experimental results also match with the DFT defects calculation for n- and p-type Mg_2Si: no change is seen for p-type and a decrease in carrier concentration is observed for n-type.

Obtaining the carrier concentration from the Seebeck coefficient

Supplementary Figure S6 shows the dependence of the Seebeck coefficient with carrier concentration (Pisarenko plot) according to the single parabolic band model (SPB) [2]. In this model, the transport properties are obtained following the equations given by:

$$|S| = \frac{k_B}{e} \times \left(\frac{2F_1(\eta)}{F_0(\eta)} - \eta\right)$$

$$n = 4\pi \left(\frac{2m_d^* k_B T}{h^2}\right)^{1.5} F_{\frac{1}{2}}(\eta)$$

Where $\eta = \frac{E_F}{k_B T}$ is the reduced chemical potential, m_d^* the density of states effective mass and n the carrier concentration, k_B is the Boltzmann's constant, $F_i(\eta)$ is the Fermi integral of order i. The value of $2.1\,m_0$ was taken for the effective mass after analyzing the transport properties of the material.

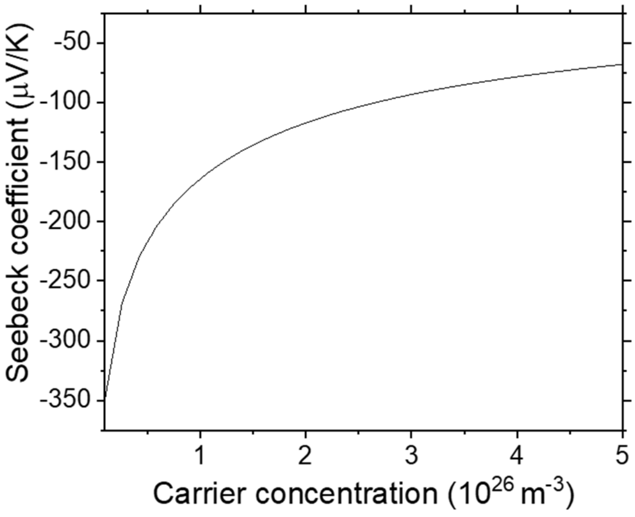

Figure S6. Pisarenko plot for n-type $Mg_2(Si,Sn)$, with an effective mass of $2.1m_0$.

References

1. Ayachi, S.; Deshpande, R.; Ponnusamy, P.; Park, S.; Chung, J.; Park, S.; Ryu, B.; Müller, E.; de Boor, J. On the relevance of point defects for the selection of contacting electrodes: Ag as an example for

Mg_2(Si,Sn)-based thermoelectric generators. Mater. Today 2021, 16, 100309, doi:10.1016/j.mtphys.2020.100309.2. Kamila, H., Prashant S., Sankhla, A., Yasseri, M., Pham, H.N., Dasgupta, T., Mueller, E., de Boor, J., Analyzing transport properties of p-type Mg_2Si–Mg_2Sn solid solutions: optimization of thermoelectric performance and insight into the electronic band structure. *J. Mater. Chem. A* **2019**, *7*, 1045–1054.
